# BpOmpW antigen administered with CAF01 adjuvant stimulates comparable T cell responses to Sigma adjuvant system

**DOI:** 10.1016/j.jvacx.2024.100438

**Published:** 2024-01-13

**Authors:** Julen Tomás-Cortázar, Conor Quinn, Niamh Corcoran, Alfonso Blanco, Dennis Christensen, Siobhán McClean

**Affiliations:** a UCD Conway Institute, University College Dublin, Belfield, Dublin 4, Ireland; b School of Biomolecular and Biomedical Science, University College Dublin, Dublin, Ireland; c Center for Vaccine Research, Statens Serum Institut, Copenhagen S, Denmark

**Keywords:** Vaccine, Adjuvant, Melioidosis, T cell responses, Interferon γ, Intracellular pathogen, Cellular immunity, Cationic adjuvant formulation

## Abstract

There are no licensed vaccines to protect vulnerable populations from the potentially fatal tropical infection, melioidosis, despite its causative agent, *Burkholderia pseudomallei,* being endemic in tropical and subtropical regions. A promising vaccine candidate, BpOmpW protected mice from melioidosis infection for up to 81 days and stimulated robust interferon gamma responses in CD4^+^, CD8^+^, NK and NKT cells. In order to progress to human studies, selection of an adjuvant with an acceptable human safety profile that stimulates appropriate correlates of protection is essential. Here we demonstrate that the CAF01 vaccine adjuvant elicits optimal immune correlates of protection when administered with our BpOmpW vaccine. Specifically, we demonstrate that CAF01 administered with BpOmpW elicits robust Th1 responses, with potent IFN-γ responses in CD4^+^ and CD8^+^ T cells and NKT cells, in addition to Th17 and Th2 responses. This formulation will be particularly effective in protecting susceptible populations including people with type 2 diabetes from melioidosis.

## Introduction

Melioidosis is a serious, often fatal tropical infection caused by the intracellular pathogen, *Burkholderia pseudomallei* [1], [2]*.* This soil and water-borne bacterium is transmitted via aerosols, ingestion, or skin abrasions. It is endemic in southeast Asia and Northern Australia and a recent modelling framework analysis predicted that *B. pseudomallei* is ubiquitous throughout the tropics and sub-tropics [3]. Moreover, it is likely that melioidosis is severely underreported in the 45 countries in which it is endemic and is likely be endemic in a further 34 countries [3]. Indeed, a recent CDC Health Advisory (July 2022) has indicated that melioidosis is also endemic in areas of the Mississippi Gulf Coast and persists in the environment in Texas [4]. It is the most common cause of community-acquired bacteraemia and the third most common cause of death from infectious disease in Thailand [5]. The case fatality rates vary from 35 % to 42 % in Thailand to 26 % recorded in Australia [2] and the predicted mortality is 89,000 deaths annually, comparable to that of measles and higher than dengue fever (12,000 deaths p.a. WHO) [3]. People with diabetes mellitus (DM) have a 12-fold increased risk of melioidosis and experience more severe disease [2], which is a concern as DM is becoming more prevalent in regions where melioidosis is endemic [6], [7].

The vaccine antigen, BpOmpW, stimulated extended protection with 75 % of mice surviving a lethal *B. pseudomallei* challenge for up to 81 days [8] which is the longest demonstrated protection for a melioidosis vaccine to date. The Sigma-adjuvant system (SAS)-adjuvanted BpOmpW vaccine stimulated effector CD4^+^ and CD8^+^ T cells and CD4^+^ CD25^+^ Foxp3^+^ regulatory T cells, and specifically stimulated interferon gamma (IFN-γ) responses in CD4^+^ and CD8^+^ T cells, natural killer (NK), and natural killer T (NKT) cells [9]. It is well recognised that interferon γ (IFN-γ) responses are particularly important to protect people from melioidosis [2], [10], [11]. Recent studies have identified that melioidosis survivors showed elevated CD4^+^ and CD8^+^ T-cell mediated IFN-γ responses to *B. pseudomallei* relative to those that died from the infection [10]. Moreover, CD4^+^ T cells contributed to half the protective responses in melioidosis survivors, while natural killer cells (NK cells) contributed 23 % of the IFN-γ protective response in this cohort [10], [11].

Tailoring of optimal vaccine responses relies on having specific antigens with an appropriate safe adjuvant that promotes specific immune profiles which protect against the infecting pathogen. SAS, composed of a squalene emulsion incorporating the TLR-4 ligand, monophosphoryl Lipid A, and trehalose dicorynomycolate (a C-type lectin mincle receptor ligand) is a highly effective adjuvant [8] but does not have a human safety record. In contrast, alum, which is widely used in licenced vaccines and which elicits robust humoral immunity, offered zero protection of mice when used as an adjuvant with BpOmpW, highlighting the importance of selecting the appropriate adjuvant [8]. Thus selecting an adjuvant with a human safety profile that stimulates an appropriate robust protective cellular immune responses is critical to protecting people with and without diabetes. We selected a vaccine adjuvant with a proven human safety profile, CAF01 (Statens Serum Institut) [12], [13]. It is a liposomal adjuvant composed of N,N’ dimethyl-N,N’ dioctadecylamonium (DDA), combined with the synthetic α,α’-trehalose-6,6′-dibehenate, another mincle receptor agonist [14] with demonstrated efficacy in models of other intracellular pathogens, including TB and malaria [13], [15], [16]. Moreover, it elicited robust cellular Th1/Th17 responses and antigen-specific humoral responses in a novel vaccine against *Chlamydia trachomatis* which also protected against genital chlamydia infection [17], [18]. In the current study, we have immune-profiled the responses of mice immunised with CAF01-adjuvanted BpOmpW and show that CAF01 stimulates robust CD4^+^, CD8^+^, NK T cell responses, matching the immune fingerprint correlating with protection against melioidosis.

## Results

### CAF01 produced moderate BpOmpW-specific antibody responses

**Fig. 1:**
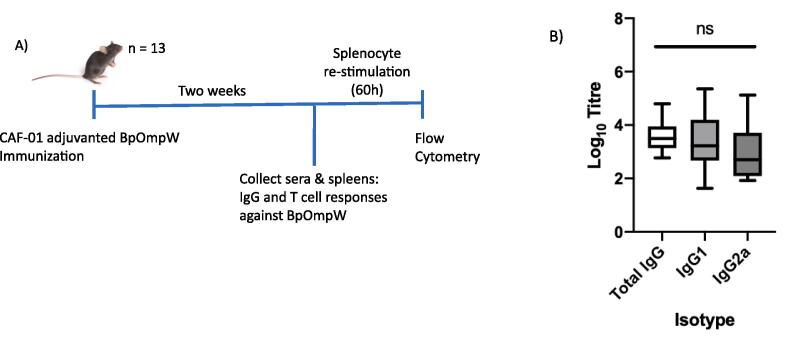
A) Schematic illustration of the mouse immunisation and sampling timeline. B) Comparison of BpOmpW specific total IgG, IgG1 and IgG2a end point titres in sera of BpOmpW immunised mice with CAF01 as adjuvant. Titres were calculated as the inverse of the lowest dilution at which OD_450_ was greater than twice the standard deviation of the CAF01 only treated control sera. Statistical analysis was performed using a one-way ANOVA.

Groups of 13 C57BL/6J mice were immunized once with CAF01-adjuvanted recombinant BpOmpW or with CAF01 alone and sera collected two weeks later (Fig. 1, A). Serological analysis of the mouse sera following immunization showed moderate total IgG, IgG1 and IgG2a seroconversion within two weeks of a single immunisation with titre values of 3.55, 3.40 and 2.93, respectively (Fig. 1, B). The IgG2a/IgG1 ratio was indicative of a mixed Th1/Th2 response, with no statistically significant differences between any of the isotypes measured.

### CAF01-adjuvanted BpOmpW activated and elicited T cell effector and regulatory T cells

**Fig. 2:**
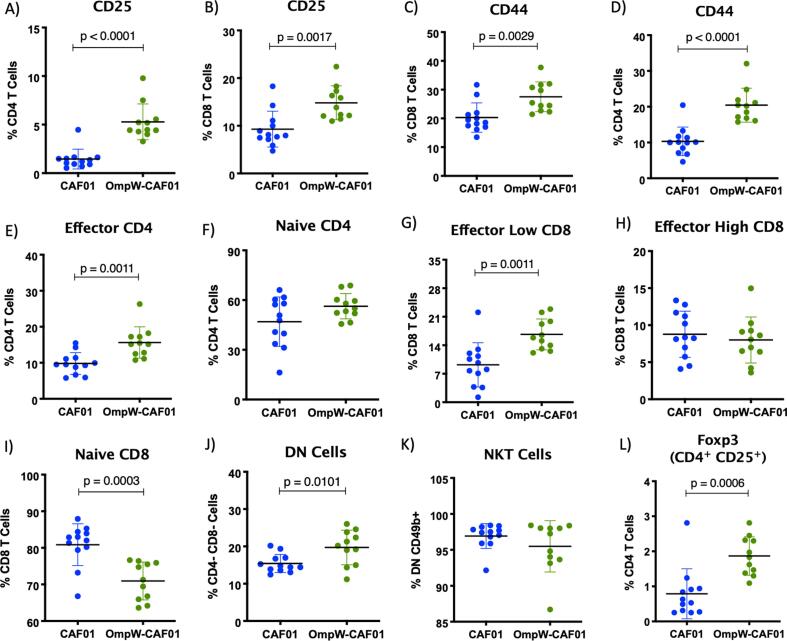
**BpOmpW immunisation with CAF01 as adjuvant activated CD4^+^ and CD8^+^ T cell populations.** (A-D) Percentages of CD4^+^ (A) and CD8^+^ (B) T cells expressing CD25 and CD44 activation markers. Percentages of effector and naïve populations of CD4^+^ and CD8^+^ T cells defined by relative levels of CD45RB and CD44, (E) effector CD4^+^ T cells: CD45RBlow CD44high; (F) naïve CD4^+^ T cells: CD45RBhigh CD44low; (G) effector CD45RBhigh CD44high CD8^+^ T cells; (H) effector CD8^+^ T cells CD45RBlow CD44high and (I) naïve CD8^+^ T cells CD45RBhigh CD44low. (J) Percentages of Double Negative (DN) T cells as CD4^-^ CD8^-^. (K) Percentages of NKT cells as CD4^-^ CD8^-^ CD49b^+^. (L) Percentages of regulatory T cells or Tregs as CD4^+^ CD25^+^ Foxp3^+^. The p values represent the statistical following two-tailed t-tests.

To examine the influence of CAF01 on antigen-specific T cell responses, splenocytes of immunised mice or adjuvant-only treated mice were re-stimulated with BpOmpW *in vitro* and T cell activation and cytokine responses measured by flow cytometry. Levels of the IL-2 receptor CD25 were greater in both CD4^+^ and CD8^+^ T cells relative to the CAF01 only control group (Fig. 2, A–B; p < 0.0001 and p = 0.0017, respectively). In addition, CD44 activation markers were significantly higher in CD4^+^ and CD8^+^ cells compared to the control group (Fig. 2, C-D; p < 0.0029, p < 0.0001, respectively). To further examine T cell activation, we also analysed the effector subsets in CD4^+^ and CD8^+^ T cells. CD45RBlo CD44hi CD4^+^ effector T cells were significantly increased in the CAF01-adjuvanted BpOmpW group relative to control (Fig. 2, E; p = 0.0011), while naïve CD4^+^ T cells, CD45RBhi CD44lo, remained comparable (Fig. 2, F). BpOmpW adjuvanted with CAF01 increased the long-term CD45RBhi CD44hi CD8^+^ effector T cell population compared tocontrol (Fig. 2, G; p = 0.0011). In contrast, CD45RBlo CD44hi short-term CD8^+^ effector T cells remained comparable to the control group (Fig. 2, H). CD8^+^ naïve T cells were significantly reduced in the CAF01-adjuvanted BpOmpW group relative to control (Fig. 2, I; p = 0.0003). Similarly, CD4 and CD8 double negative (DN) cells were more abundant in the CAF01-adjuvanted BpOmpW immunised group relative to the CAF01 control group (p = 0.0101; Fig. 2, J). CD49b^+^ NKT cells constituted nearly all these DN cells (Fig. 2, K). Finally, CD4^+^CD25^+^Foxp3^+^ regulatory T cells were increased in response to CAF01-adjuvanted BpOmpW relative to the adjuvant-only controls (Fig. 2, L; p = 0.0006).

### CAF01-adjuvanted BpOmpW elicited cytokine responses in CD4^+^, CD8^+^ and NKT cells

**Fig. 3:**
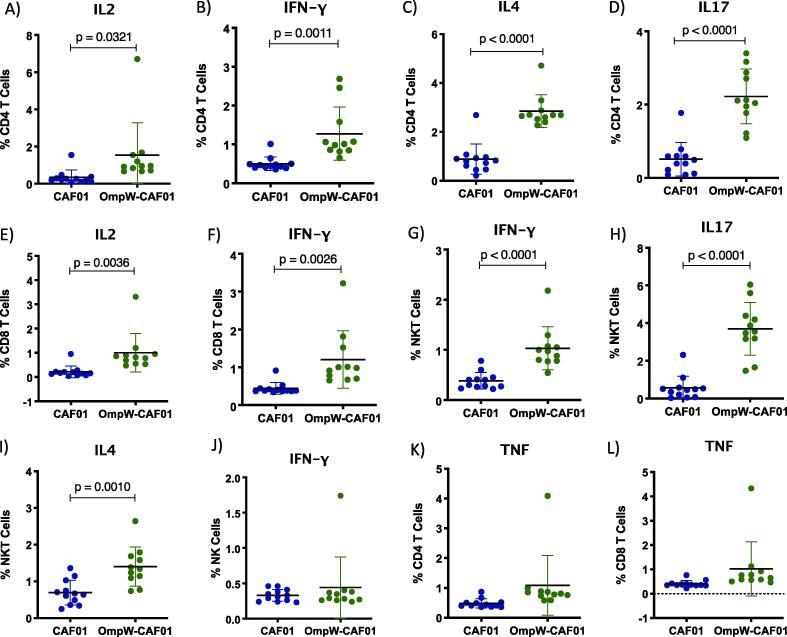
**BpOmpW immunisation with CAF01 as adjuvant stimulated IFN-g responses**. (A-D) Percentages of CD4^+^ T cells expressing IL-2 (A), IFN-γ (B), IL-4 (C) and IL-17 (D). (E-F) Percentage of CD8^+^ T cells expressing IL-2 (E) and IFN-γ (F). G-I) Percentages of NKT cells expressing IFN-γ (G), IL-17 (H) and IL-4 (I). J) Percentages of Natural Killer (NK) cells expressing IFN-γ. K-L) Percentages of CD4^+^ (K) and CD8^+^ (L) T cells expressing TNF. The levels of significance are represented as p values from a two-tailed *t*-test.

To determine whether the elevated effector T cell numbers were also reflected in cytokine responses, we measured intracellular cytokine responses. IL-2 levels were significantly higher in the CAF01-BpOmpW group in both CD4^+^ and CD8^+^ T cells compared to the adjuvant alone control (Fig. 3, A–B; p = 0.0321, p = 0.0036, respectively). Moreover, it was apparent that Th1, Th2 and Th17 responses, as determined by IFN-γ, IL-4 and IL-17 levels respectively, were significantly elevated in the presence of the CAF01-adjuvanted antigen relative to the controls (Fig. 3, C-E; p = 0.0011, p < 0.0001 and p < 0.0001, respectively). IFN-γ expressing CD8^+^ T cells were also more abundant following CAF01-BpOmpW immunisation (Fig. 3F; p = 0.0026).

Furthermore, mice immunised with CAF01-adjuvanted BpOmpW showed enhanced IFN-γ, IL-4 and IL-17 responses in NKT cells in comparison with the adjuvant only group (Fig. 3, G–I; p < 0.0001, p < 0.0001, p = 0.0010, respectively). IFN-γ-producing CD49b^+^ NK cells in the BpOmpW/CAF01 group remained comparable to the control group after a single immunisation (Fig. 3, J). TNF responses remained comparable to adjuvant control in both CD4^+^ and CD8^+^ T cells from CAF01-BpOmpW immunized mice (Fig. 3; K, L).

**Fig. 4:**
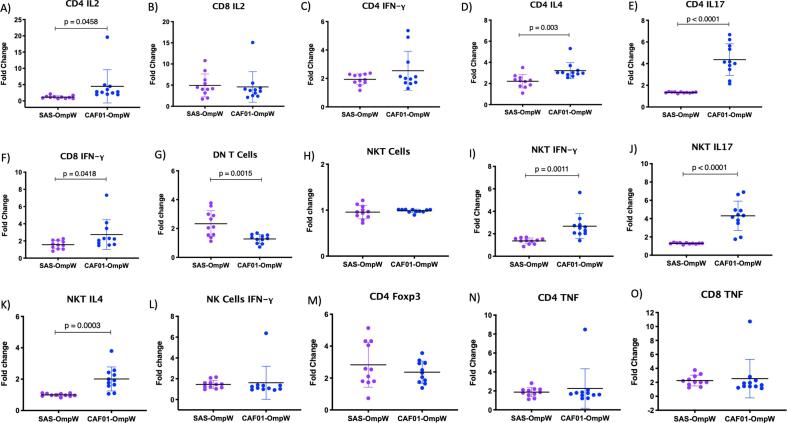
**Comparison of CAF01-adjuvanted responses with previous SAS-adjuvanted responses in BpOmpW immunised mice.** (A-B) Fold change in CD4^+^ (A) and CD8^+^ (B) T cells expressing IL-2 cytokine. C-E) Fold change in CD4^+^ T cells expressing IFN- γ (C), IL-4 (D) and IL-17 (E). D) Fold change in CD8^+^ T cells expressing IFN- γ;.G) Fold change in Double Negative (DN) cells (CD4^-^ CD8^-^). H) Fold change in Natural Killer T cells (NKT). I-K) Fold change in NKT cells expressing IFN- γ (I), IL-17 (J) and IL-4 (K). L) Fold change in Natural Killer (NK) cells expressing IFN- γ; M) Fold change in Regulatory T cells (Tregs). N-O) Fold change in CD4^+^ (N) and CD8^+^ (O) T cells expressing TNF. Asterisks denote statistically significant differences according to a two-tailed *t*-test. The levels of significance are represented as follows: (*, p < 0.05); (**, p < 0.01,); (***, p < 0.001); (****, p < 0.0001).

In order to compare CAF01 immune responses with those observed in previous immune-profiling studies on SAS adjuvant [8], [9], we determined the fold changes in immune markers from both SAS- [9] and CAF01-adjuvanted BpOmpW groups relative to their respective control groups’ average (SAS alone, CAF01 alone). In consideration of the 3Rs, the data from two independent studies on the same BpOmpW antigen batch were compared, rather than repeat immunisations testing both adjuvants in parallel. In order to compare between individual experiments, we determined the fold-changes in responses elicited by the respective antigen-adjuvant immunisation relative to adjuvant only. The CAF01 stimulated significantly stronger IL-2 responses in CD4^+^ T cells relative to SAS (Fig. 4, A; p = 0.0458), while IL-2 expressing CD8^+^ T cells remained comparable (Fig. 4, B). The CD4^+^ T cell IFN-γ response was comparable to that of SAS (Fig. 4, C), while IL-4 and IL-17 cytokines were more abundant in CAF01-adjuvanted BpOmpW immunised mice (Fig. 4, D-E; p = 0.003 and p < 0.0001). Interestingly, IFN-γ expressing CD8^+^ T cells were stronger in mice immunised with BpOmpW + CAF01 compared to BpOmpW + SAS (Fig. 4, F; p = 0.0418). This is a key finding as IFN-γ CD8^+^ cell populations were very low in every fatal melioidosis case examined by Jenjaroen *et al.* [10]. Although DN cells showed more robust responses in SAS relative to CAF01-adjuvanted mice immunised with BpOmpW (Fig. 4, G; p = 0.0015), the latter also showed a significant DN response. This is crucial due to the altered immune profile of diabetes mellitus patients. DN T cells are linked to melioidosis survival in people with T2D as low levels of DN T cells were demonstrated in diabetes patients that died [19]. A key finding is that IFN-γ, IL-4 and IL-17 responses in NKT cells were greatly enhanced in BpOmpW/CAF01 immunised mice compared to mice immunised with BpOmpW/SAS (Fig. 4, I-K; p = 0.0011, p < 0.0001 and p = 0.0003).

**Fig. 5:**
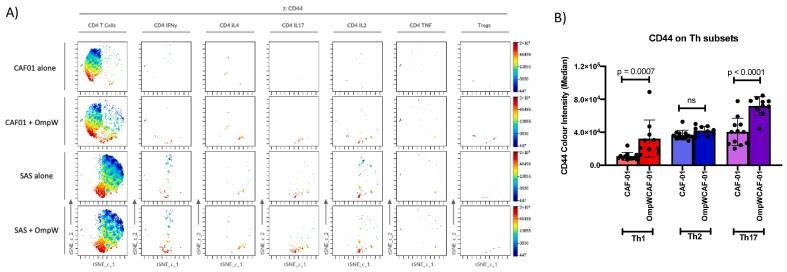
**Dimensionality reduction of CD4^+^ T cell population by tSNE-CUDA analysis. A)** Representative tSNE-CUDA plots of CAF01 alone, CAF01 + OmpW, SAS alone and SAS + OmpW groups depicting the density of CD4^+^ T cell population, Th1 cells (CD4 IFN-y), Th2 cells (CD4 IL-4), Th17 cells (CD4 IL-17), TNF expressing CD4^+^ T cells (CD4 TNF), IL-2 expressing CD4^+^ T cells and regulatory T cells or Tregs. The amount of the activation marker CD44 is represented using the colour spectrum as z-axis. B) Medians of colour intensity of CD44 in IFN-y-expressing CD4^+^ T cells or Th1, IL4-expressing CD4^+^ T cells or Th2 and IL17-expressing CD4^+^ T cells or Th17. Statistical analysis was performed using one-vay ANOVA and p-values presented.

To further analyse the activation and cytokine expression of the different T helper populations in both adjuvants across the 14 colours used, we performed a dimensionality reduction analysis on CD4^+^ T cells. The tSNE-CUDA clearly showed that, in addition to IL2 and Tregs, both adjuvants supported increases in the Th1, Th2 and Th17 populations in the presence of BpOmpW (Fig. 5), with Th1 and Th17 populations being the most activated T helper populations as determined by the expression of CD44. We also evaluated the activation of different T helper subsets as determined by the intensity of the CD44 activation marker (Fig. 5, A). Consistent with immune correlates of protection for melioidosis, Th1 and Th17 populations showed increased expression of the CD44 activation marker in the BpOmpW + CAF01 immunised group relative to the CAF01 only treated group (p = 0.0007 and p < 0.0001, respectively) while the Th2 population was unchanged (Fig. 5, B).

## Discussion

The prevalence of melioidosis in endemic countries, its recent emergence in tropical and sub-tropical regions, together with the increased risk for people with diabetes which is increasing in prevalence in these countries highlights the urgent need to have an effective vaccine to protect vulnerable populations. Several melioidosis vaccine candidates have been examined, including subunit vaccines such as Hcp1 and AhpC [20], [21]; gold-linked nano-particle protein constructs [22]; outer membrane vesicle (OMVs) [23], [24] and live attenuated vaccines [20], [25], [26], but the BpOmpW antigen has shown uniquely extended protection over 81 days when adjuvanted with SAS [8]. Progressing this promising candidate to human studies is a critical stage in its development. Selecting a suitable, safe adjuvant is vital to its evaluation in humans, however there is a limited choice of adjuvants available with existing human safety data. We selected CAF01 as a potential suitable adjuvant as it demonstrated robust antigen-specific Th1/Th17 profile in pre-clinical studies and its safety has been demonstrated in humans [12], [13], [15], [16], [27], [28]. Specifically, CAF01 induces durable and potent CD4^+^ T cells in mice and humans [16], [27], [29], [30], [31]. Robust T-cell responses are essential for an effective melioidosis vaccine, as lower *B. pseudomallei*-specific T-cell responses were associated with increased mortality in melioidosis patients; while CD4^+^, CD8^+^-interferon γ-producing cells were associated with survival [10]. Overall, we show that CAF01 elicited comparable immune correlates of protection relative to the SAS adjuvant that we had used previously.

NKT cells can contribute early resistance to infection through IFN-γ production and have been shown to contribute to early splenic IFN-γ responses in a mouse melioidosis model [32]. DN NKT cells activated by *Mycobacterium tuberculosis* infected antigen presenting cells have been suggested to generate memory responses and protective immune responses in Hepatitis B infection [33], [34] and we speculate that this response may contribute to protection against melioidosis. Finally, IFN-γ expressing NK cells, regulatory CD4^+^ T cells and TNF in CD4^+^ and CD8^+^ T cells remained comparable in both adjuvants (Fig. 4, L–O).

A recent systems biology approach comparing CAF01 with three other clinically tested adjuvants, including the TLR4 receptor ligand, GLA-SE and alum, showed that CAF01-activated T follicular helper cell populations in draining lymph nodes with frequencies comparable across the other three adjuvants tested [35]. Germinal centre B cells were also identified at high frequencies in CAF01 group [35]. In contrast, while the TLR-4 ligand GLA-SE stimulated strong humoral responses after one immunisation, CAF01 and Alum induced significant responses after only two or more immunisations. This supports our moderate serological responses after single immunisations of mice with CAF01-adjuvanted BpOmpW.

A limitation of our study is that both adjuvants were not compared in the same experiments. The SAS-adjuvanted BpOmpW analysis had been previously thoroughly examined [9] and in line with the principles of the 3Rs we could not justify repeating this analysis on more animals. Furthermore, the ability for CAF01 adjuvanted BpOmpW to protect animals against *B. pseudomallei* challenge remains to be confirmed, availability of facilities to test Class 3 pathogens is a complication for these kinds of experiments.

Overall, we have shown that BpOmpW/CAF01 provides comparable, or for some cell populations, superior immune correlates to those of BpOmpW/SAS and thus, given its safety profile, represents a suitable adjuvant for human studies. In addition, recent studies have shown that CAF01 induced robust mucosal immunity when used to prime subsequent oral immunisations in the absence of adjuvant [28]. Ultimately a heterologous prime/boost regime may be the optimal means of protecting patients from a pathogen such as *B. pseudomallei* that can be transmitted by inhalation and oral ingestion in addition to penetration via skin abrasions, which further strengthens the potential for CAF01 as an appropriate adjuvant for protection against melioidosis.

## Material and methods

### Ethics statement

All work involving animals was approved by University College Dublin Ethics Committee (AREC-19–13-McClean), and mice were maintained according to the regulations of the Health Products Regulatory Authority (Directive 2010/63/EU and Irish Statutory Instrument 543 of 2012) with the Authorisation number AE18982/P166.

### Immunisation of C57BL/6J mice for immunophenotyping

The recombinant BpOmpW used in all experiments was produced and provided by Lionex GmBH in 20 mM Ammonium bicarbonate. Male C57BL/6J mice were given free access to food and water and subjected to a 12 h light/dark cycle. Groups of C57BL/6J male mice were immunized subcutaneously with 50 μg BpOmpW or saline mixed 1:1 in a final volume of 100 μL with CAF01 or with CAF01 plus saline as negative control. Two weeks later, mice were humanely killed by sedation with isoflurane followed by CO_2_ exposure before exsanguination by cardiac puncture for serological analysis. Spleens were also processed for splenocyte restimulation with the vaccine antigen.

### Determination of BpOmpW-specific IgG isotypes by ELISA

Microtitre plates were coated with purified BpOmpW in sodium bicarbonate buffer (pH 9.4) at 4 °C overnight, the coating solution removed, and plates blocked with 10 % FBS solution in PBS at room temperature for 1 h. Serum samples were serially diluted (5-fold) in PBS containing 10 % FBS and antigen-specific antibodies measured in triplicate using anti-mouse IgG, IgG1 or IgG2a conjugated antibodies (ab97023, ab97240, ab97245, respectively from Abcam) as described previously [9].

### Splenocyte restimulation with BpOmpW antigen and flow cytometry analysis

Splenocytes were extracted from the spleens using Ammonium-Chloride-Potassium (ACK) lysing buffer to remove red blood cells. One million cells per well were plated in 10 % FBS RPMI medium with antibiotics on 96-well-plates and stimulated for 60 h with 50 μg/mL BpOmpW. Five hours before harvesting the cells, 5 μg/mL Brefeldin A was added to increase the accumulation of intracellular cytokines. Cells were collected by centrifugation and stained for flow cytometry as described previously [9]. Briefly, the cells were incubated with Fc Block (anti-CD16/CD32; BD BioSciences) for 5 min on ice and labelled with Viakrome 808 (Beckman Coulter) and fluorochrome-labelled antibodies against CD4, CD49b, CD45RB, CD8a, CD25, CD44 and CD3 surface markers (BD Biosciences) for 30 min on ice. Intracellular IL-2, IL-4, IFN-γ, IL-17, IL-9, TNF, and Foxp3 (BD Bioscience) were analysed with a BD Cytofix/CytoPerm^TM^/Fixation/ Permeabilization Solution Kit (BD Biosciences) according to manufacturer instructions. The antibodies were titrated for optimal performance and Beckman Coulter Versacomp beads were used for compensation. A Beckman Coulter CytoFLEX LX (NUV full configuration) was used for the analysis of the samples with the gains for each one of the markers optimised by performing a gaintration. The Quality Control of the instrument was performed using the Beckman Coulter Daily QC beads and IR Daily QC as per manufacturer specifications. Fluorescence Minus One control (FMOs) were used for every single marker in order to generate the gates and several FMOs were used daily for verification purposes. The gating strategy and corresponding FMOs are shown in Figure S1-S7. Data analysis was performed with Beckman Coulter CytExpert v. 2.4. The presentation of the plots was made in log transformation (both axis). Samples, replicates or parental cells with low events were discarded to avoid rare cases. For this reason, two mice were discarded from the analysis for CAF-01 + BpOmpW datasets and one mouse from the CAF01 control dataset. The dimensionality reduction (tSNE-CUDA) analysis of the CD4^+^ T cell populations was performed by Cytobank (Beckman Coulter) using all events (3,349,680) from 130 FSC files. Iteration number was set as automatic (2,233) with a perplexity of 30 and a thetha value of 0.5. The supplemental file shows the full MIFlowCyt: The Minimum Information about a Flow Cytometry Experiment.

### Statistical analysis

Results are presented as means ± SE unless otherwise stated and differences between groups were analysed using a T-test using GraphPad Prism, version 7. A p-value < 0.05 was considered statistically significant.

### CRediT authorship contribution statement

**Julen Tomás-Cortázar:** Formal analysis, Investigation, Methodology, Writing – original draft, Writing – review & editing, Validation, Visualization. **Conor Quinn:** Formal analysis, Investigation, Methodology. **Niamh Corcoran:** Data curation, Formal analysis, Methodology. **Alfonso Blanco:** Methodology, Resources, Validation. **Dennis Christensen:** Conceptualization, Writing – review and editing. **Siobhán McClean:** Conceptualization, Formal analysis, Investigation, Writing – original draft, Writing – review and editing, Funding acquisition, Supervision.

## Declaration of competing interest

The authors declare the following financial interests/personal relationships which may be considered as potential competing interests: [Dennis Christensen reports a relationship with State Serum Institute that includes: employment. Siobhán McClean and Julen Tomás Cortázar have patent #GB2307810.8 pending to University College Dublin. There are no other relationships to declare If there are other authors, they declare that they have no known competing financial interests or personal relationships that could have appeared to influence the work reported in this paper].

## Data Availability

Data will be made available on request.
