# Supplementary material for: BpOmpW antigen administered with CAF01 adjuvant stimulates comparable T cell responses to Sigma adjuvant system
Source: Vaccine X. 2024 Jan 13;17:100438. doi: 10.1016/j.jvacx.2024.100438 (PMC10831100; doi:10.1016/j.jvacx.2024.100438)
Supplement: Supplementary data 1 [file mmc1.docx]

1. **Experiment Overview**

**Purpose:** The purpose of the experiment is to characterize T cell responses against BpOmpW vaccine antigen using CAF-01 adjuvant

**Keywords:** Splenocytes, vaccine, BpOmpW, antigen, T cell responses, CAF-01

**Experiment Variables:** Percentage of splenocytes exposed to BpOmpW antigen

**Organization:** Flow Cytometry Core at UCD Conway Institute. University College Dublin, Belfield, Dublin 4, Ireland. Eircode: D04 V1W8

**Primary Contact:** Julen Tomás Cortázar ([julen.tomascortazar@ucd.ie](mailto:julen.tomascortazar@ucd.ie))

**Date:** Cytometry performed on February 22, 2020.

**Conclusions:** The BpOmpW antigen using CAF-01 as adjuvant induce the necessary T cell responses to combat melioidosis disease.

**Quality control measures:** FMOs were done for each marker. Splenocytes collected from CAF-01 only adjuvanted mice as negative cells in order to provide a staining control.

1. **Flow Sample/Specimen Details**

**Sample Material Description:** Splenocytes from immunized C57Bl/6j mice were stained with a panel antibody in 96wp. Mice were 6-8 weeks old. All mice were male immunized with CAF-01 adjuvant alone or CAF-01 + BpOmpW, 13 mice in each group.

**Sample Characteristics:** After lysing red blood cells from the mashed spleens, remaining splenocytes were stained for T cell markers and intracellular cytokines. One million of cells were plated in vitro and stimulated with BpOmpW antigen for 60 hours.

**Sample Treatment Description:** One million of splenocytes resuspended in 100ul PBS 1% FBS. Block Immunoglobulin FcReceptor. Purified Rat anti-Mouse BD FcBlockTM, CD16/CD32 (BD Pharmingen 553142) have been used to block nonspecific staining due to FcR. Cells were washed with PBS 1% FBS before using BD FcBlock. To block FcR, cells were incubated with one microliter BD FcBlock in 100ul PBS 1% FBS cell suspension for 5 minutes on ice in 96wp. Cells were washed and incubated with a panel of antibodies for 30 minutes on ice using 25ul PBS 1% FBS + 25ul Brilliant Stain Buffer from BD Horizon (563794). For intracellular staining, BD Cyofix/Cytoperm kit (BD 554714) was used. 100ul Cytofix/Cytoperm buffer were used for 20 minutes on ice to permeabilize and fix the cells in 96wp. Splenocytes were then intracellularly stained in 25ul Brilliant Stain Buffer + 25ul 1X Perm/Wash bufer in 96wp. Cells were washed twice with 200ul 1X Perm/Wash buffer and leave overnight in this buffer. Finally, next day, splenocytes were resuspended in PBS 1% FBS for flow cytometry analysis. All centrifugations were done at 300g for 5 min.

**Fluorescence Reagent Description:** Each sample has been stained and assigned to a Laser/channel according to the following table.

| **LASER/CHANNEL** | **Fluorophore-Antibody.** |
| --- | --- |
| B525-FITC | FITC Rat Anti-Mouse CD8a (BD Pharmingen 553030) |
| B690-PC5.5 | BB700 Rat Anti-Mouse TNF (BD Horizon 566510) |
| Y585-PE | PE Armenian Hamster anti-Mouse IL-9 (BD Pharmingen 561463) |
| Y763-PC7 | PE-Cy7 Rat Anti-Mouse CD25 (BD Pharmingen 552880) |
| R660-APC | Alexa Fluor 647 Rat anti-Mouse Foxp3 (BD Pharmingen 560401) |
| R712-APCA700 | APC-R700 Rat Anti-Mouse CD44 (BD Horizon 565480) |
| R763-APCA750 | APC Cy7 Rat Anti-Mouse CD3 (BD Pharmingen 560690) |
| V450-PB | BV421 Rat Anti-Mouse IL-2 (BD Pharmingen 562969) |
| V525-KrO | BV510 Rat Anti-Mouse CD45RB (BD OptiBuild 740107) |
| V610 | BV605 Rat Anti-Mouse IL-4 (BD Horizon 564007) |
| V660 | BV650 Rat Anti-Mouse IFN-y (BD Horizon 563854) |
| V763 | BV786 Rat Anti-Mouse IL-17A (BD Horizon 564171 |
| NUV405 | BUV395 Rat Anti Mouse CD4 (BD Horizon 563790) |
| NUV525 | BUV563 Hamster Anti-Mouse CD49b (BD OptiBuild 741280) |
| IR885 | ViaKrome 808 Fixable Viability Dye (Beckman Coulter C36628) |

1. **Instrument Details**

**Instrument Manufacturer:** Beckman Coulter: <https://www.beckmancoulter.com/>

**Instrument Model:**

CytoFlex LX. Model No B90883.

Serial Number BA34050.

**Instrument configuration and settings:**

Flow Cell and Fluidics: The instrument has not been altered; fixed-alignment cuvette flow cell.

Light Sources: The instrument has not been altered.

Optical Filters: The instrument has not been altered.

Optical detectors: The instrument has not been altered.

The following figure shows the filter and detector configuration:


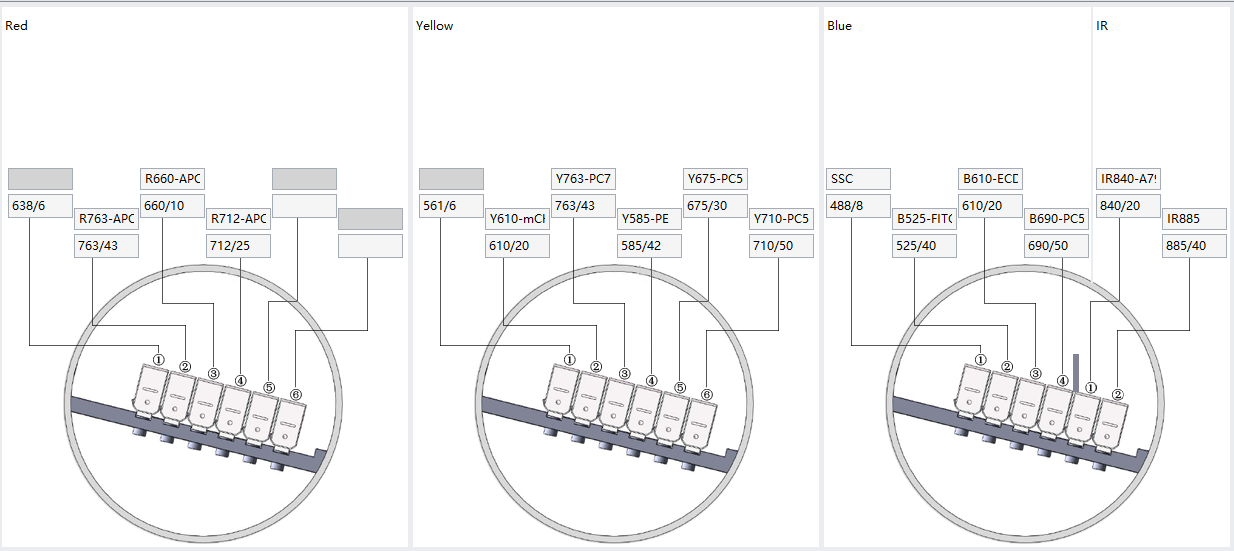


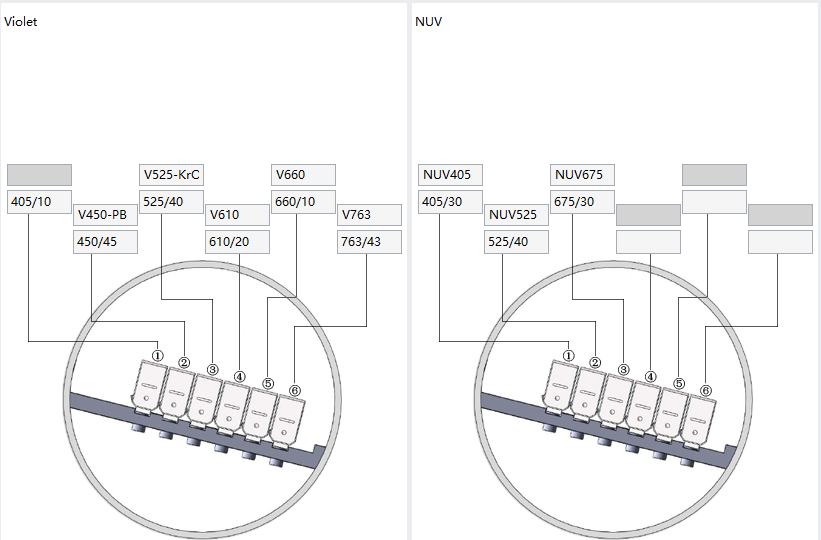


1. **Data Analysis Details**

**List-mode Data Files**: FCS data files can be obtained by contacting Dr Julen Tomás Cortázar or Assoc Prof. Siobhán McClean.

**Data Transformation Details:**

Purpose of Data Transformation: Visualization and gating.

Data Transformation Description: Cytexpert software

**Compensation Description:**
