# Supplementary figures and images for: BpOmpW antigen administered with CAF01 adjuvant stimulates comparable T cell responses to Sigma adjuvant system
Source: Vaccine X. 2024 Jan 13;17:100438. doi: 10.1016/j.jvacx.2024.100438 (PMC10831100; doi:10.1016/j.jvacx.2024.100438)

## Slide 1
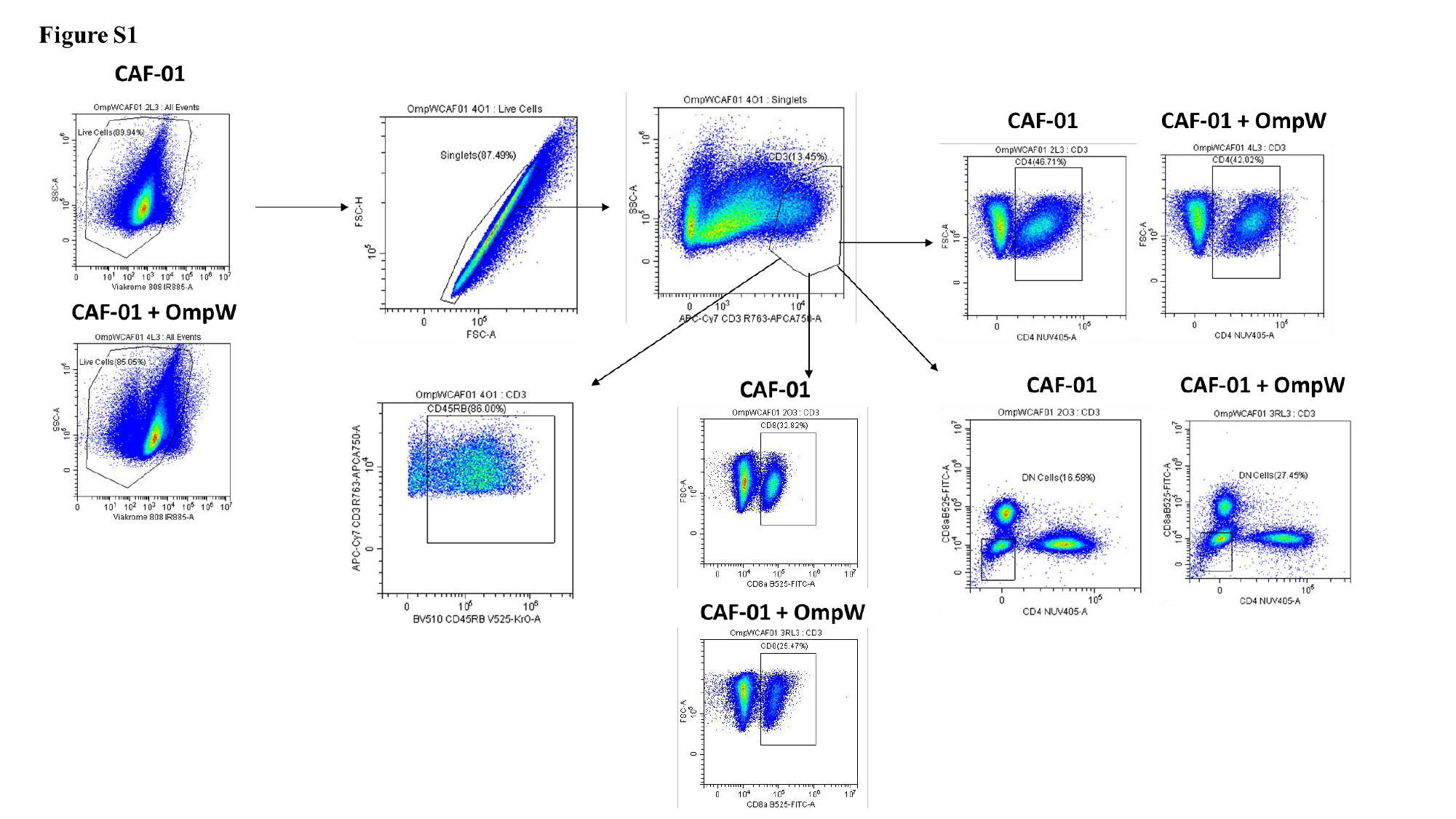

## Slide 2
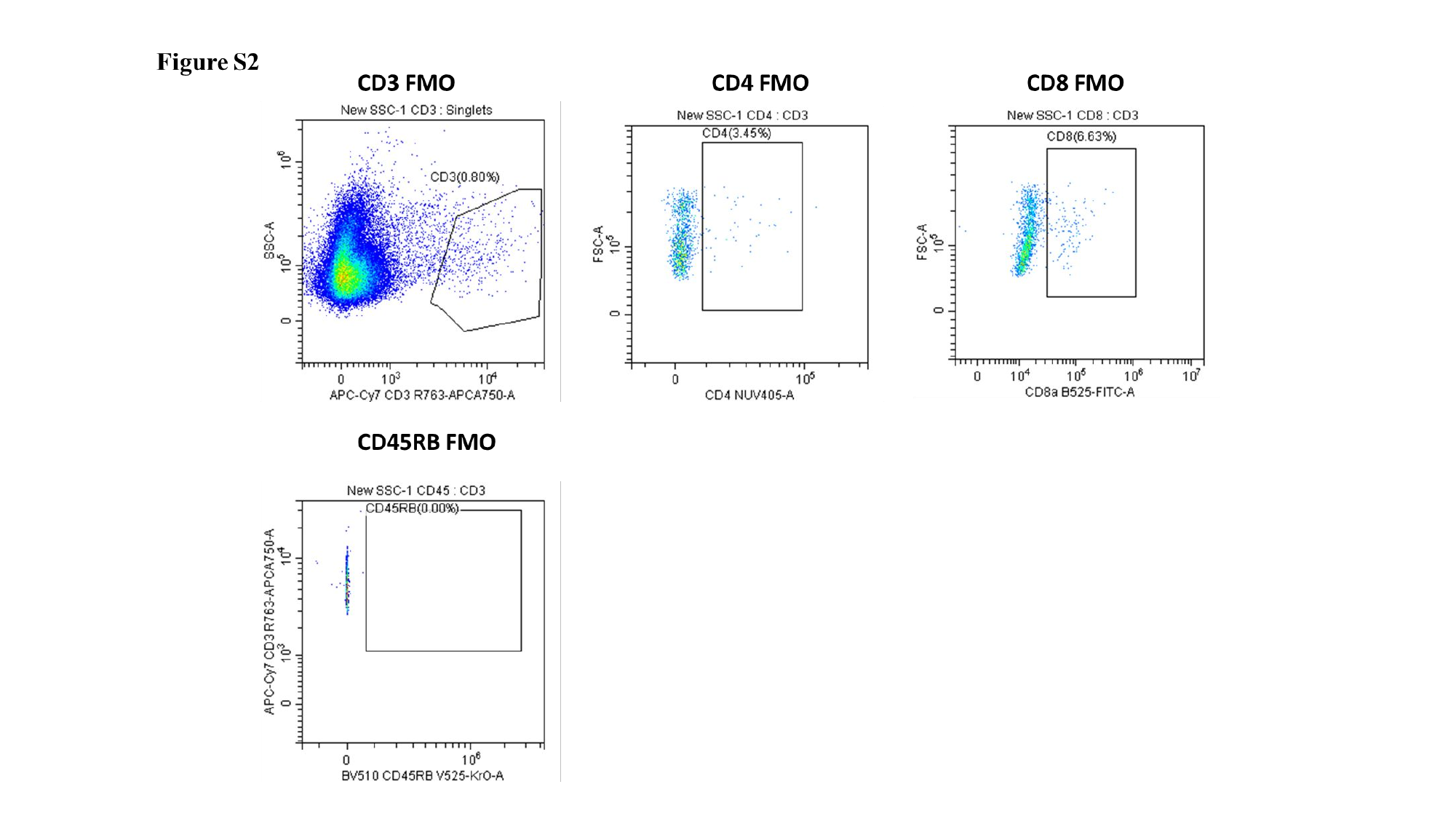

## Slide 3
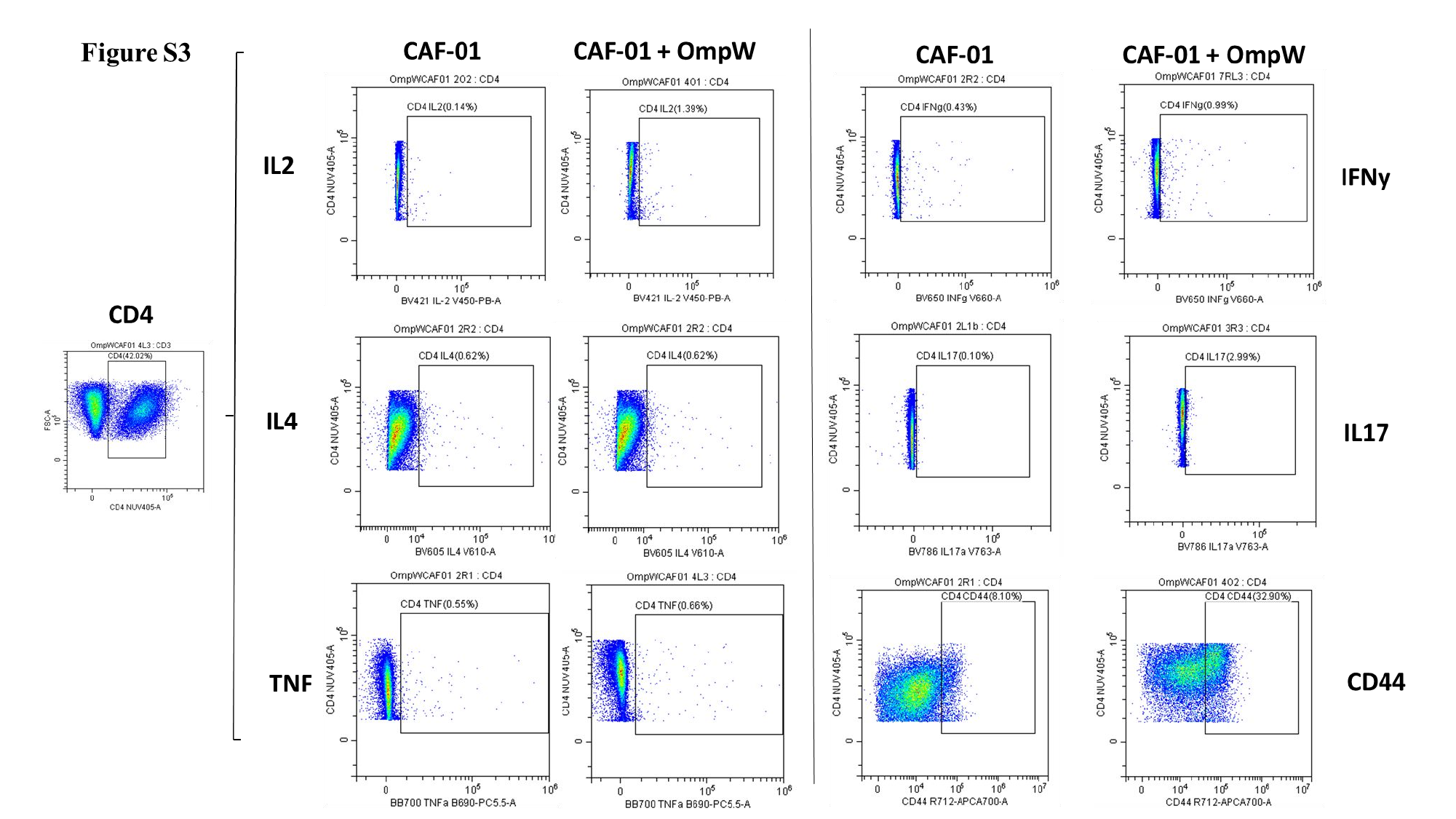

## Slide 4
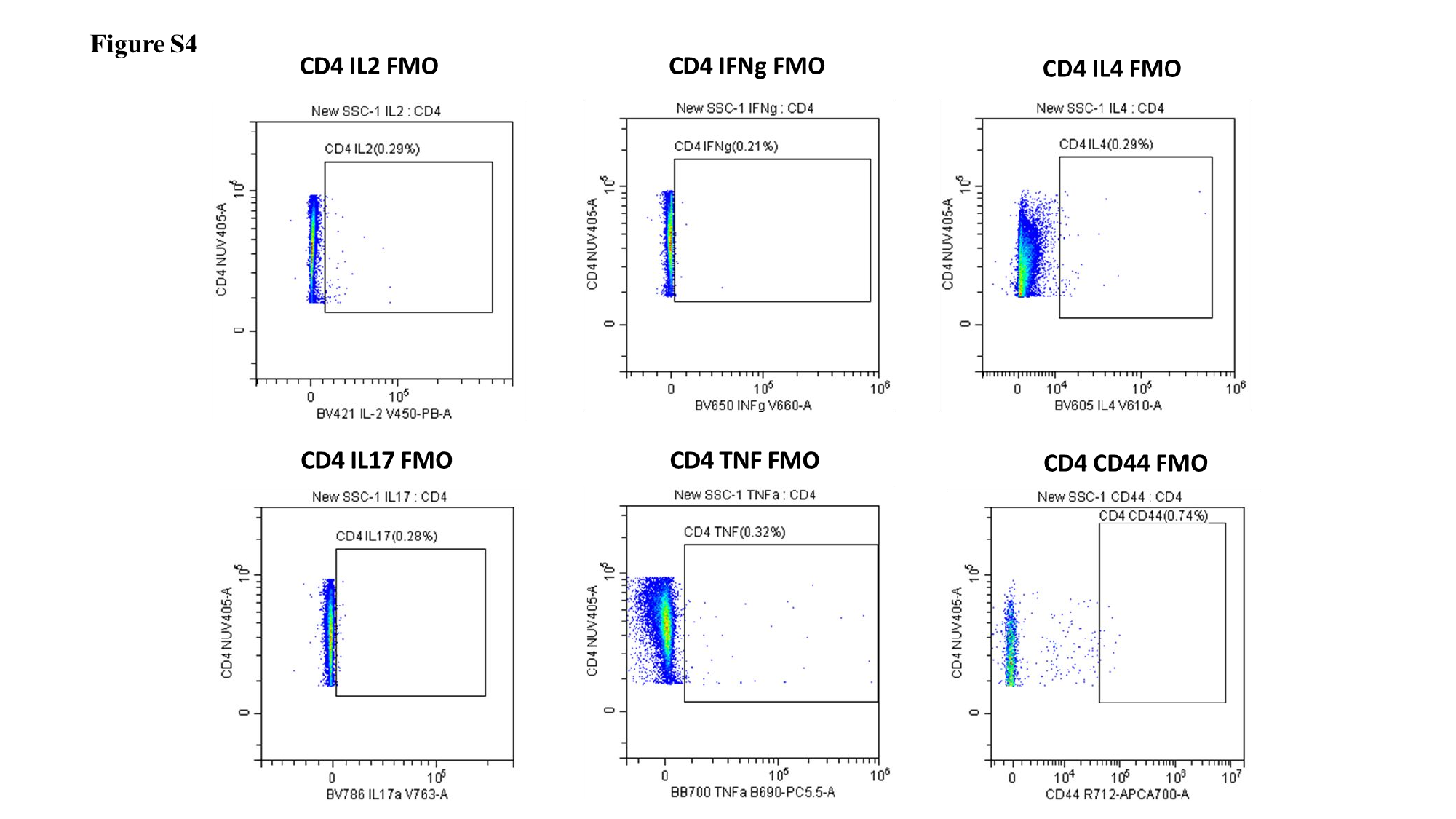

## Slide 5
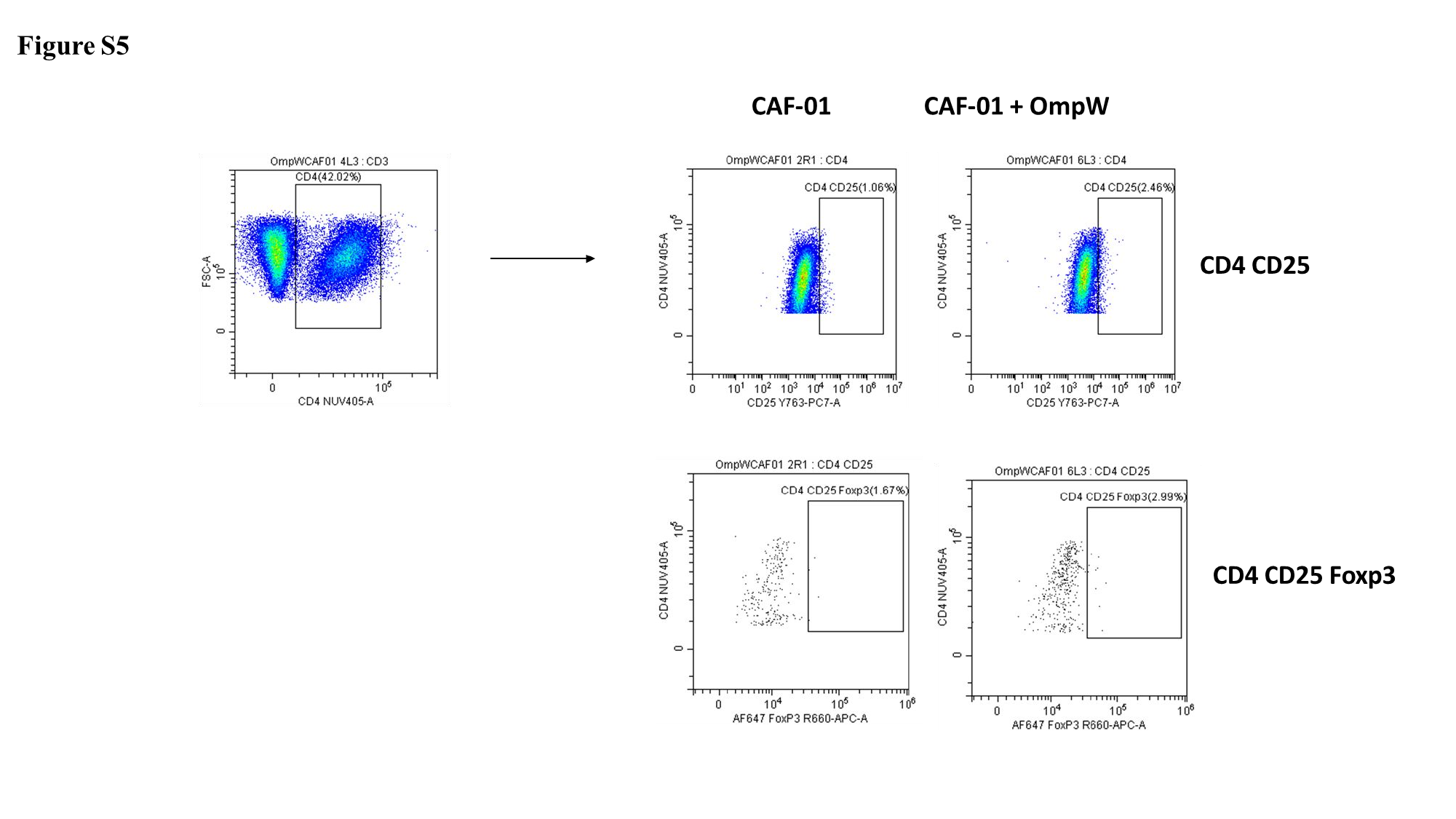

## Slide 6
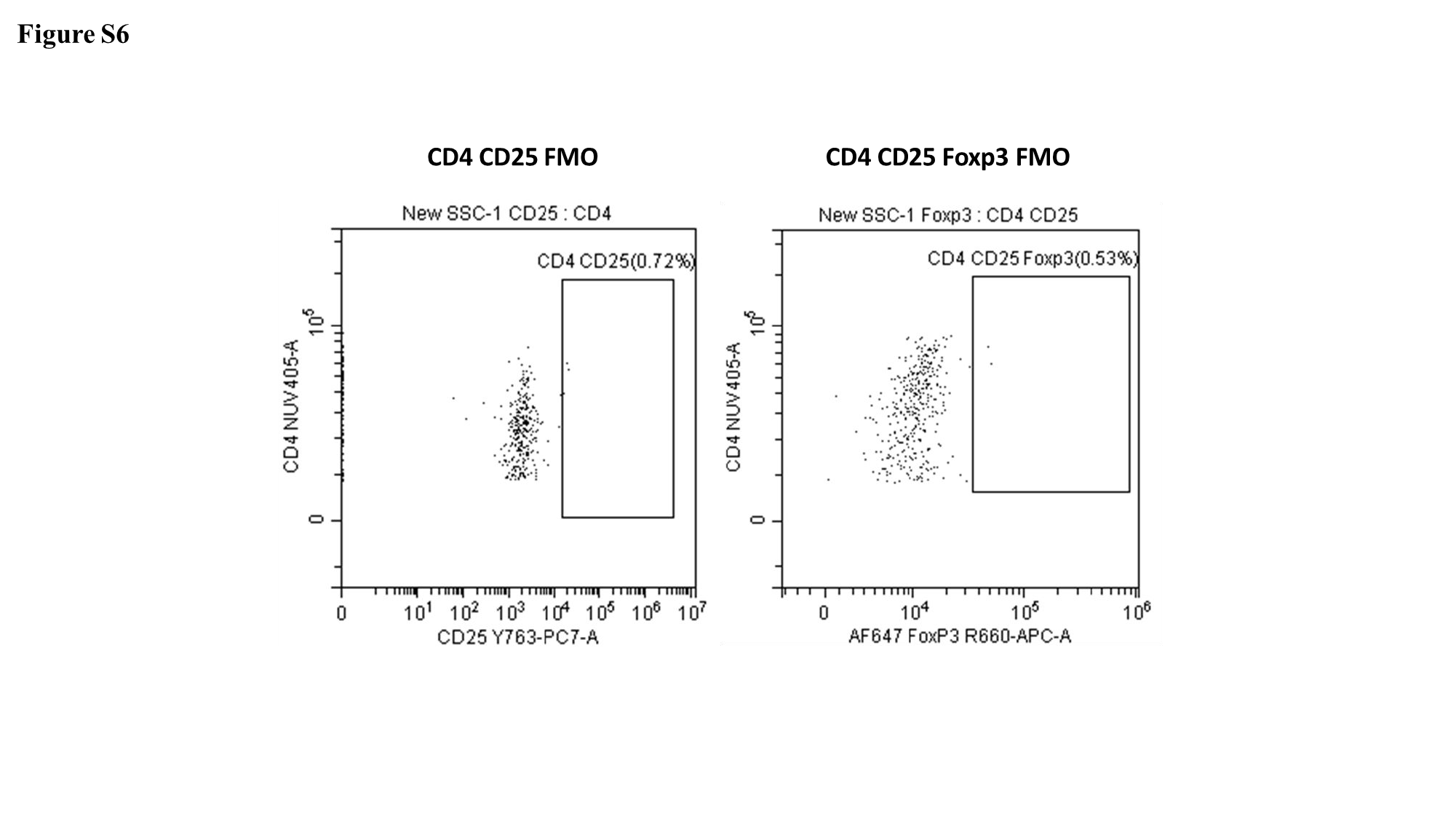

## Slide 7
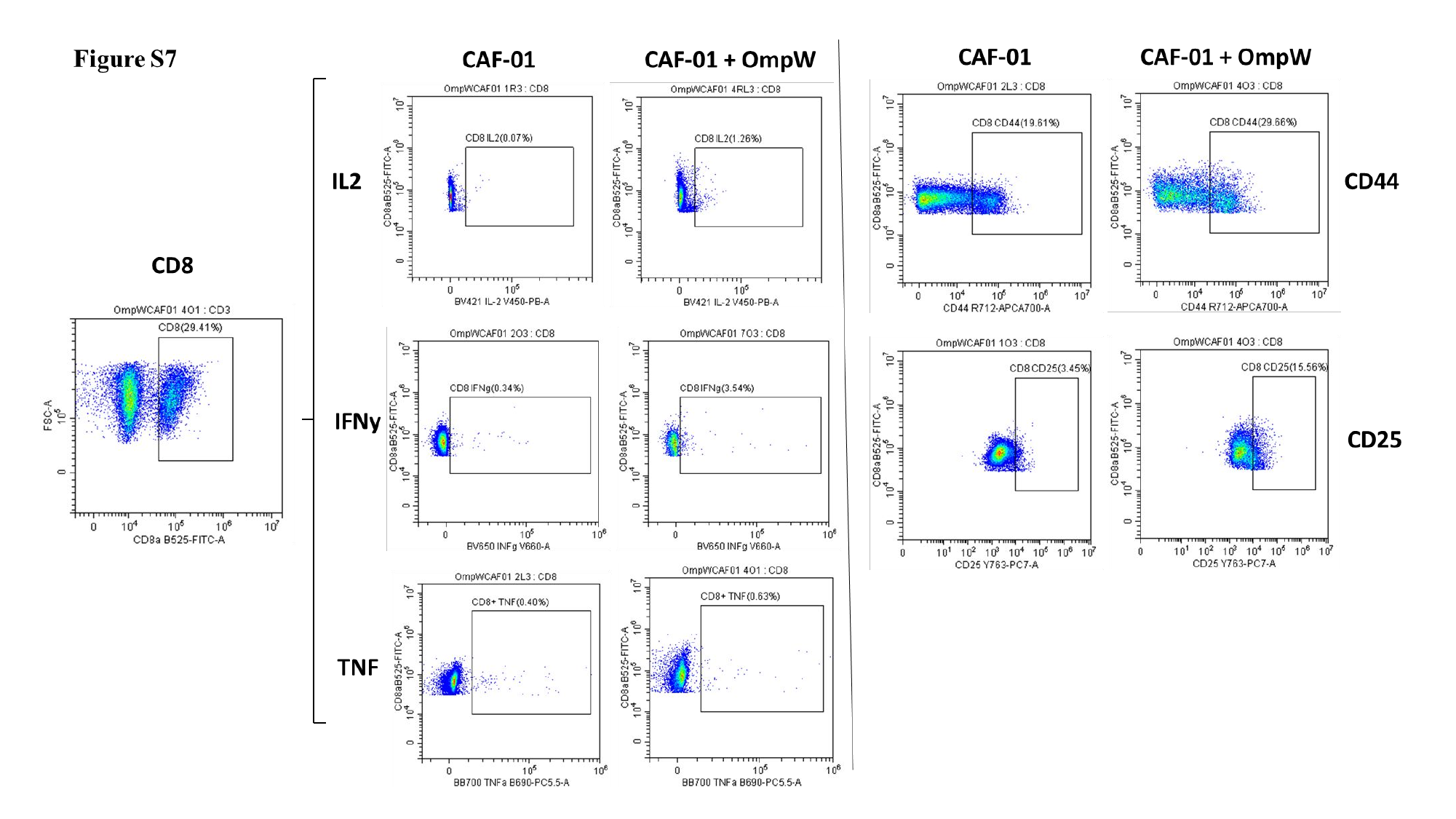

## Slide 8
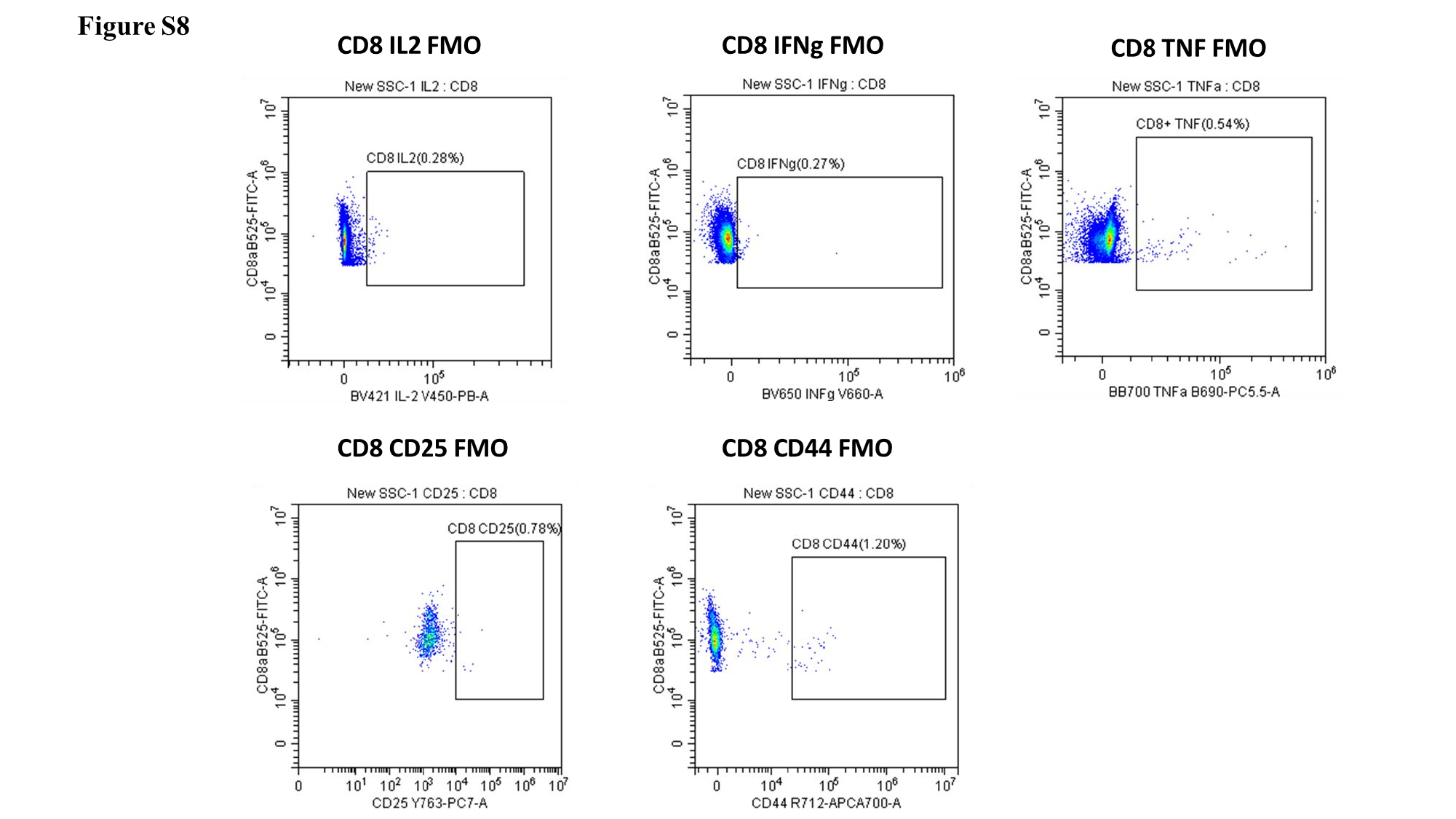

## Slide 9
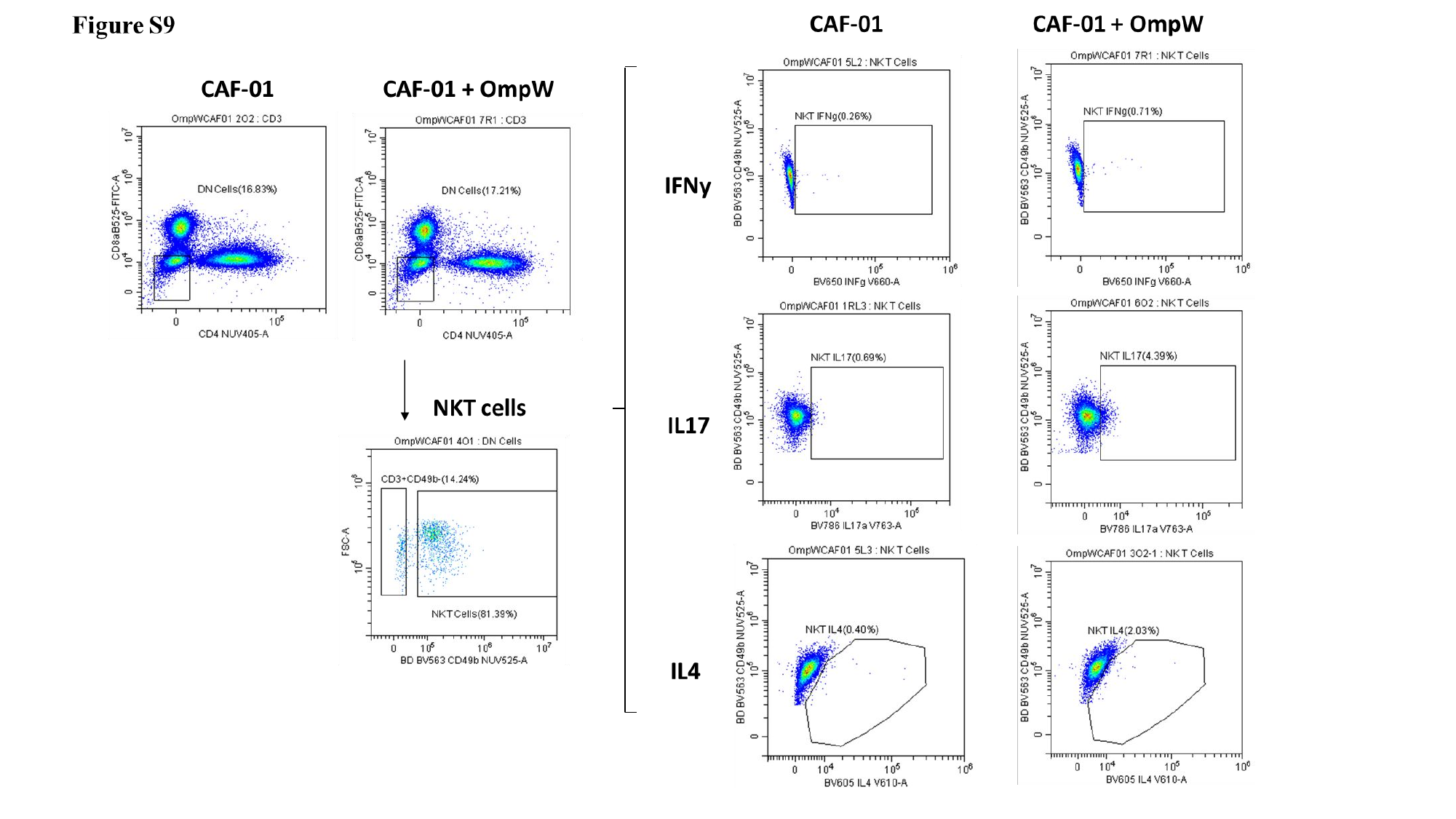

## Slide 10
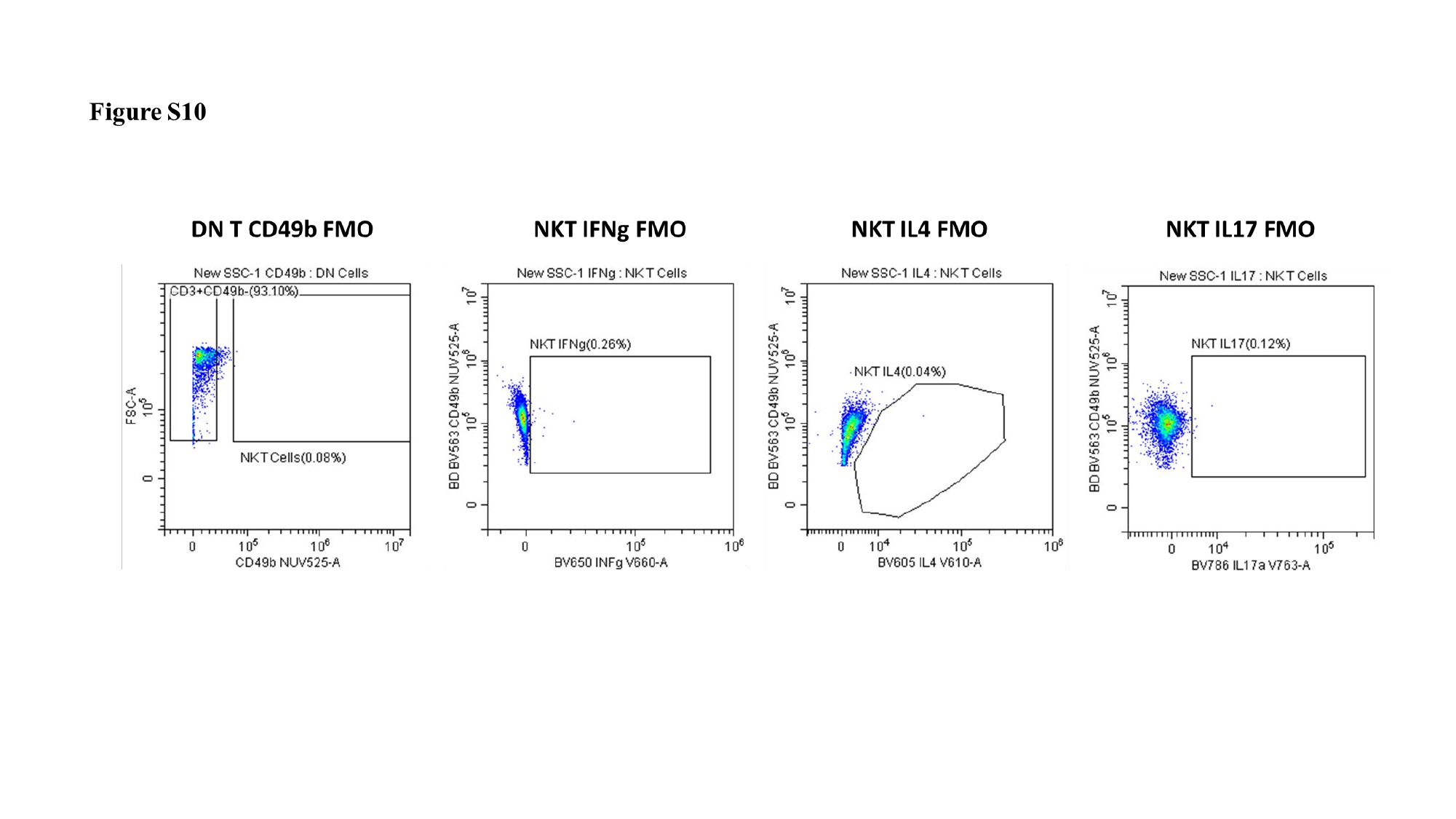

## Slide 11
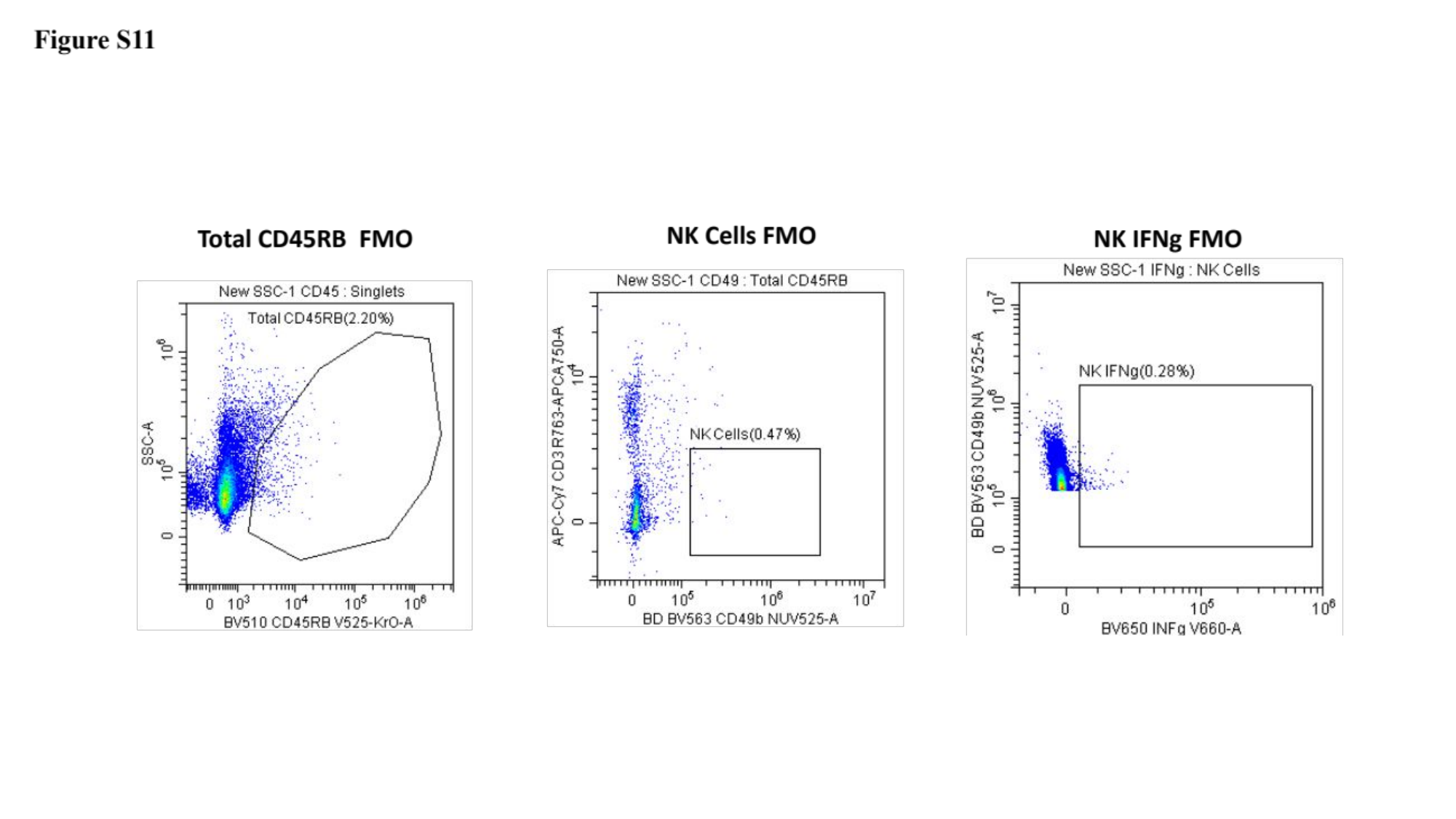

## Slide 12
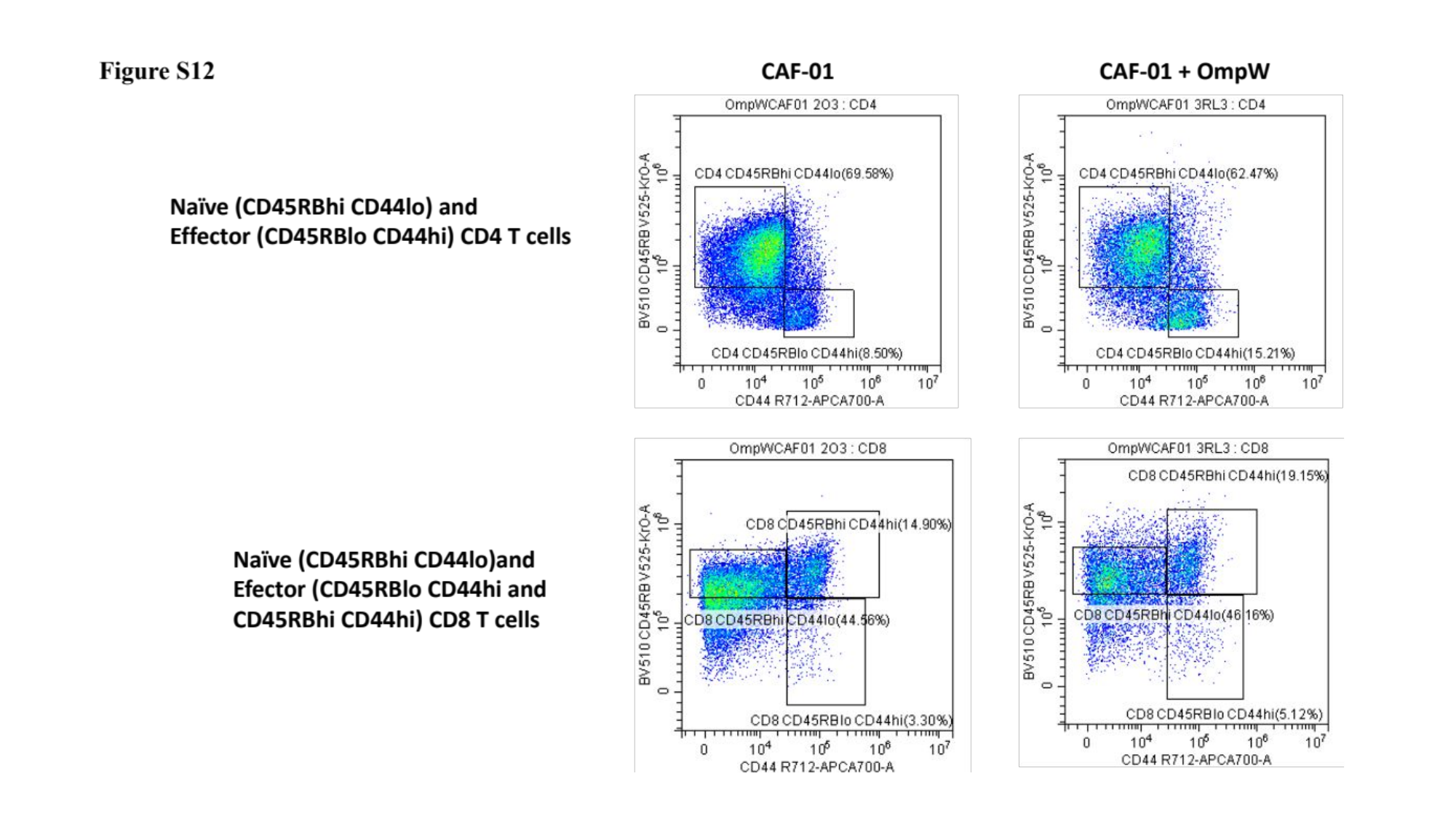

## Slide 13
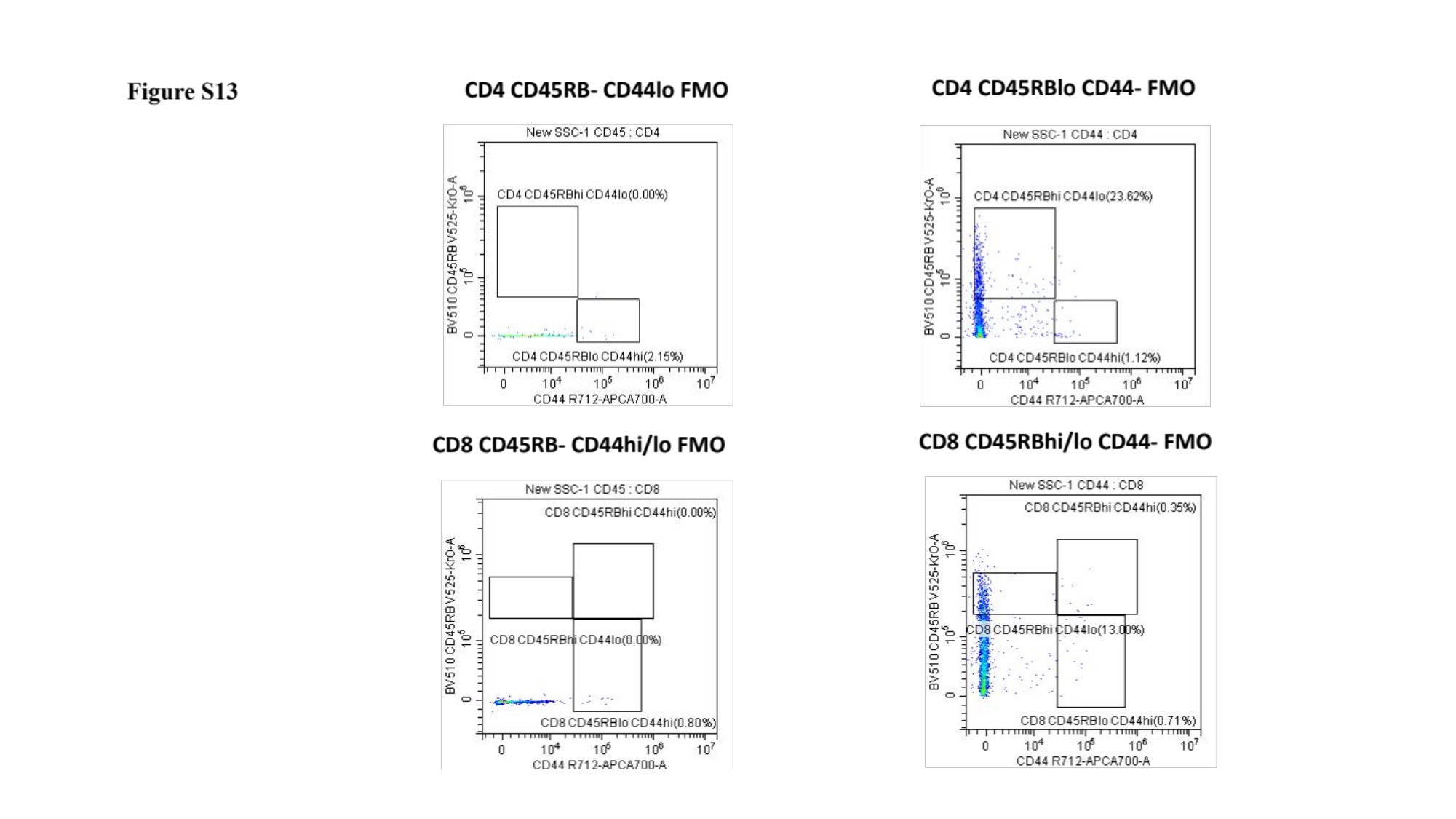

Supplement: Supplementary data 2 [file mmc2.pptx]
